# Robot usability in the wild: bridging accessibility gaps for diverse user groups in complex forestry operations

**DOI:** 10.1007/s10209-025-01234-2

**Published:** 2025-06-13

**Authors:** Florian Ehrlich-Sommer, Bernhard Hörl, Christoph Gollob, Arne Nothdurft, Karl Stampfer, Andreas Holzinger

**Affiliations:** 1 Human-Centered AI Lab, Institute of Forest Engineering, Department of Ecosystem Management, Climate and Biodiversity, University of Natural Resources and Life Sciences Vienna, Vienna, Austria; 2 Institute of Forest Growth, Department of Ecosystem Management, Climate and Biodiversity, University of Natural Resources and Life Sciences Vienna, Vienna, Austria

**Keywords:** Robot usability, Forest robotics systems, LiDAR scanning technology, System usability testing, Human–robot interaction, Usability evaluation methods, User-centered design, Operational robotics efficiency

## Abstract

This study evaluated the usability and effectiveness of robotic platforms working together with foresters in the wild on forest inventory tasks using LiDAR scanning. Emphasis was on the Universal Access principle, ensuring that robotic solutions are not only effective but also environmentally responsible and accessible for diverse users. Three robotic platforms were tested: Boston Dynamics Spot, AgileX Scout, and Bunker Mini. Spot’s quadrupedal locomotion struggled in dense undergrowth, leading to frequent mobility failures and a System Usability Scale (SUS) score of 78 ± 10. Its short, battery life and complex recovery processes further limited its suitability for forest operations without substantial modifications. In contrast, the wheeled AgileX Scout and tracked Bunker Mini demonstrated superior usability, each achieving a high SUS score of 88 ± 5. However, environmental impact varied: Scout’s wheeled design caused minimal disturbance, whereas Bunker Mini’s tracks occasionally damaged young vegetation, highlighting the importance of gentle interaction with natural ecosystems in robotic forestry. All platforms enhanced worker safety, reduced physical effort, and improved LiDAR workflows by eliminating the need for human presence during scans. Additionally, the study engaged forest engineering students, equipping them with hands-on experience in emerging robotic technologies and fostering discussions on their responsible integration into forestry practices. This study lays a crucial foundation for the integration of Artificial Intelligence (AI) into forest robotics, enabling future advancements in autonomous perception, decision-making, and adaptive navigation. By systematically evaluating robotic platforms in real-world forest environments, this research provides valuable empirical data that will inform AI-driven enhancements, such as machine learning-based terrain adaptation, intelligent path planning, and autonomous fault recovery. Furthermore, the study holds high value for the international research community, serving as a benchmark for future developments in forestry robotics and AI applications. Moving forward, future research will build on these findings to explore adaptive remote operation, AI-powered terrain-aware navigation, and sustainable deployment strategies, ensuring that robotic solutions enhance both operational efficiency and ecological responsibility in forest management worldwide.

## Introduction and motivation

The advancement of robotic systems has significantly transformed various industries, offering innovative solutions for tasks that traditionally demanded extensive human labor. In the field of forest engineering, where precision, efficiency, and adaptability are paramount, integrating robotic systems holds the potential to revolutionize operations. However, effective deployment hinges on the usability and practicality of these systems in real-world scenarios [1]. This study builds upon prior research conducted in controlled environments, where the usability of different robotic types was evaluated with forest engineering students. While these initial findings provided valuable insights, they lacked the complexity and unpredictability of real-world forest settings [2]. Working directly with users on robots enables the possibility of fostering human–robot collaboration, which will become an important factor as AI algorithms continue to grow more capable in the future [3].

The current study addresses this gap by shifting the focus to actual forest environments—an important step, as the Universal Access (UA) concept is already reinforced through legislation [4]. Future forest engineers were given the opportunity to interact directly with three distinct robotic systems, each designed to enhance specific tasks in forestry. Participants were not only trained to operate these systems but were also encouraged to document their experiences, highlighting potential pitfalls, shortcomings, and areas for improvement. This dual approach—hands-on interaction combined with reflective feedback—ensures that the findings are grounded in both practical application and critical evaluation. Testing with novices in the technology ensures that usability can be assessed effectively for a broad range of user groups [5].

A key component of this study was the comparison between traditional human-operated LiDAR scanning and robot-assisted scanning. LiDAR technology, integral to modern forestry, requires precise execution to deliver reliable data for forest management and planning [6]. By leveraging robotic systems, this study aimed to simplify and enhance the scanning process, making it accessible even to non-experts. Robotic-based systems can have a plug and play approach taking human error out of the equation. For example, fast and long range movements like moving an arm up or to the side while scanning can affect the quality of the scan at this certain point in the scan. Additionally, the robot can be setup to completely remove the operator from the LiDAR scan and therefore reduce the work involved in later data manipulation. The robotic-based system has the potential to directly produce cleaner scans while reducing human error.

Each robotic system was evaluated based on usability, efficiency, and adaptability, with a focus on solutions that could be easily implemented in everyday forestry operations. Additionally, this work emphasizes the importance of reducing time and physical labor to improve the efficiency and ergonomics of the scanning application, showcasing the potential for robotic systems to ease physical strain and streamline workflows. This is especially important in an environment where innovative features often gain more attention than their actual functionality [7].

The performance of the different robots' maneuvering capabilities through the forest was also assessed, providing insights into their ability to navigate complex terrains [8]. Furthermore, the robots’ capacity to perform LiDAR scans was directly compared to that of human operators, highlighting both the advantages and limitations of robotic enhancements in this critical task. This comparison underscores the need for further refinement to ensure that robotic systems can meet or exceed the standards set by human expertise while maintaining simplicity and ease of use.

Beyond the immediate application in forestry, this work also provides cross-domain information, recognizing that forestry is a highly complex environment. The findings from this study have the potential to influence other industries, offering valuable lessons on robotic integration in similarly challenging settings. By exploring the intersection of robotics and forestry, this research contributes not only to the advancement of forest engineering but also to broader applications of robotics in diverse fields. This underscores the current focus on moving towards Industry 5.0, where human-centered AI and human–robot interaction play a crucial role [9–12].

The insights gained from integrating robotics into forestry can be applied to a wide range of other industries, particularly those that demand precision, adaptability, and the ability to function in complex, dynamic environments. Sectors such as agriculture [13], construction, and environmental monitoring, which share similar challenges in terms of terrain, data collection, and labor intensity, could benefit from robotic technologies developed in forestry. Additionally, industries like mining [14], logistics, and disaster response, which require advanced automation and operational flexibility, stand to gain from these advancements. The focus on reducing physical strain and improving workflow efficiency in forestry can also inform labor-intensive fields, such as warehouse automation [15] and search-and-rescue operations. The hands-on training approach used in this study further provides valuable insights for workforce development across these domains, enabling professionals to better integrate and collaborate with robotics in their daily operations.

The involvement of forest engineering students in this study paves the way for the next generation of forestry experts to familiarize themselves with novel robotic solutions. This early exposure helps them understand how such technologies can be effectively integrated into their future work as professionals. By equipping students with practical experience and critical insights, this study supports the development of a workforce that is well-prepared to harness the potential of robotics in forestry. Previous research also suggests that clearly specifying the user group provides more detailed insights into the interaction [16].

The results of this research are expected to provide actionable recommendations for improving the design and deployment of robotic systems in forestry. By understanding the nuances of human–robot interaction in dynamic, real-world settings, this study contributes to the broader goal of seamlessly integrating robotics into the field of forest engineering [17]. This integration is not only crucial for increasing efficiency but also for preparing future professionals to navigate and leverage emerging technologies in their field.

Through this study, we aim to bridge the gap between controlled experiments and practical applications, offering a comprehensive analysis of the usability and effectiveness of robotic systems in forestry. The insights gained will serve as a foundation for future innovations, ensuring that these technologies are both functional and user-friendly in the context of forest engineering.

## Background and related work

### Robot challenges in forestry

Initial tests with different robotic systems in our internal testing facility revealed potential issues that could arise when deployed in a forest environment. Various robotic platforms possess different strengths and weaknesses, some of which have already been discussed in previous studies, including their ability to overcome obstacles and maintain operational performance [2].

Although simulated environments are essential for an initial understanding of capabilities, there are limitations to the knowledge that can be gained—particularly in highly complex environments [18]. The most significant challenges that can only be assessed in the field under realistic conditions include:

- Locomotion performance on variable surfaces;
- Operational time;
- System boundaries.

This section provides an overview of these challenges to help readers understand the following experimental sections.

### Locomotion performance on variable surfaces

Locomotion is the fundamental function required for any robotic system to be effective in a given environment. Locomotion technologies range from basic wheel- and track-based robots to more advanced, animal-like designs [19, 20]. The benchmark for optimal movement in forests is the mobility of humans and animals, as their range of motion minimizes environmental impact while allowing navigation through highly complex terrains [21].

It is essential to recognize the complexity of such movement—current robotic systems, even the most advanced ones, still cannot match the efficiency of nature. Therefore, the initial useful application of robots will be restricted to the simplest and most widespread environments within forests. This includes forest roads on the less challenging end of the spectrum and areas with deadwood and regenerating vegetation on the more complex end.

Natural obstacles such as logs and deadwood might appear similar to already solved problems in everyday life, such as stair climbing or navigating piles of gravel. However, these forest obstacles present a significantly greater challenge— even climbing stairs remains difficult for most robotic systems [22]. For example, a pile of branches lacks a stable surface and presents a high risk of entanglement.

In general, locomotion methods should minimize environmental damage, as even minor disturbances can lead to significant long-term ecological issues [23]. Optimal locomotion is not only about overcoming obstacles but also doing so with minimal environmental impact. Assessing this effectively requires real-world testing with various platforms. In this study, wheeled, tracked, and legged robots will be evaluated and compared to human mobility.

### Operational time

Remote forest locations pose challenges related to operational time. Current forestry equipment relies on liquid fossil fuels such as diesel and gasoline due to their simplicity and high energy density. However, the robotics industry predominantly employs battery-electric systems, with combustion engine-powered robots being relatively rare [24].

A standard workday in forestry is eight hours, with an additional hour before and after operation for equipment maintenance, charging, data extraction, and related tasks, totaling ten hours per day. Using battery-electric robots in remote locations introduces the need for on-site electricity generation, which remains a challenge. Currently, forestry lacks sustainable charging options beyond generators or large-scale battery banks, both of which are cumbersome and reduce usability. Hybrid approaches are currently the most viable solution [25].

To maximize operational time, robots must optimize energy consumption through efficient maneuvering. As discussed in the locomotion section, humans can intuitively select optimal paths, whereas robots require complex algorithms to do the same [26]. Additionally, computing power plays a significant role in power consumption—onboard data analysis can significantly reduce battery life [27]. To address this, remote control and partial automation can reduce the computational burden while maintaining acceptable operational times [28].

### System boundaries

All systems operate within defined boundaries, and exceeding these limits often leads to failures. In forestry, various factors influence these system boundaries, including:

- Soil Composition & Inclination;
- Vegetation & Environmental Conditions;
- Task at Hand.

### Soil composition & inclination

Soil properties play a crucial role in determining the optimal robotic system for a given task. Key factors include load-bearing capacity [29] and moisture accumulation, which influence the maximum feasible size and locomotion technology [30]. For example, shifting from wheels to tracks can affect ground pressure, as seen in rut formation caused by heavy forestry machinery [31, 32].

Soil friction is another critical factor—clay-rich soils reduce traction, while gravel provides better grip. Thus, soil conditions at the operational site have a significant impact on the selection of technology [33]. Since forest soil types vary widely across Central Europe, these considerations are especially relevant [34].

Inclination is another major limitation. The ability of a system to move freely depends on slope steepness, with risks including slipping, sliding, and tipping. Slipping occurs when traction is lost due to increased inclination and can be mitigated by better traction systems or stabilizing support, such as cables. Sliding is a more extreme consequence of slipping, in which the entire vehicle loses control and descends uncontrollably [35, 36].

Tipping, the final risk factor, occurs when a vehicle loses its balance and falls over. This is more complex to predict, as it depends on the vehicle’s center of gravity and pivot point. Whether a robot moves directly up and down or traverses a slope affects its stability. Safety parameters must be set accordingly to account for all three risks, as they often occur simultaneously. Additionally, torque regulation plays a key role—sudden bursts of torque can cause any of these failures, even before reaching theoretical stability limits [37].

### Vegetation & environmental conditions

Ground-based vehicles, regardless of type, are affected by both vegetation and weather conditions. Dense tree cover limits the maximum feasible size of a robot, while young regenerating trees are highly vulnerable to damage. Running over small saplings can significantly hinder forest recovery [38].

Furthermore, vegetation changes seasonally, altering terrain accessibility. A path that is impassable in summer due to dense undergrowth may be easily navigable in winter. Weather also directly impacts traction—rain and snow reduce stability, while temperature fluctuations affect soil load-bearing capacity, sometimes leading to mass movements that render areas impassable [39].

Battery-electric systems, which dominate modern robotics, also have specific operating temperature ranges. Extreme temperatures can significantly impact battery capacity and, consequently, operational time [40].

### Task at hand

The final determining factor for a robotic system is the specific task it must perform. Key questions include the scope of the operation. For example, tree harvesting requires heavy machinery such as harvesters or chainsaws operated by humans, whereas conducting a forest inventory with a LiDAR scanner is a task suited for small-scale robots.

The considerations outlined above provide a foundational understanding for non-experts to determine suitable robotic systems for various forestry applications. Although this study focuses on forestry, the principles discussed apply to any field where robotics is deployed. Some parameters may be irrelevant in different environments, while others may become critical. For example, in industrial settings, ground conditions might be negligible, whereas human–robot interactions could be a primary concern. The interaction could also be auxiliary where the robot just helps perform a certain task and in a perfect scenario the different machines would directly know which one the user wants to operate, something that is so far only known for digital devices like computers and phones [41].

### Overall system considerations

Condensing this section down leaves one with the following set of considerations for a robot used in a forestry environment:

- Robust locomotion on a wide range of surfaces (hard packed gravel, slippery mud, soft leaves, undergrowth, etc.);
- Operational time minimum of 4 to 5 h to enable battery change in mandatory breaks but optimally the ability to operate for full 10 h with varying load to enable the operator to really be fully remote;
- Ability to fulfill the task at hand – in this case carrying a LiDAR scanner and reaching most of the forest stand to achieve a full scan (sometimes it is not even possible for the human operator to reach the entirety of a forest stand due to terrain reasons);
- Produce the least amount of damage in the forest stand. This includes the plain ground but also all the vegetation (there is always some sort of interference with the environment even by human operators, but it should be kept to a minimum).

A system will always be a compromise between all the above-mentioned, but the two key factors are always whether the robot is able to maneuver the terrain and can it fulfill the task; if these two are not doable a system deployment does not make sense from the start.

## Methods and materials

The core method behind this study was the utilization of the System Usability Scale to understand how participants interacted with the different robotic platforms, as well as the practical feasibility of using various robots in a forest environment. The robots tested included the tracked AgileX Bunker Mini, the wheeled AgileX Scout, and the Boston Dynamics Spot with quadrupedal locomotion. This practicality test encompassed not only understanding the maneuvering capabilities of these robots in a forest setting but also their ability to fulfill the task of LiDAR scanning a predefined forest area. Each testing setup will be described in the following sections, while the general parameters are outlined here.

The experimental site was located within the Rosalia Teaching Forest at an altitude of approximately 720 m above sea level. This is an experimental forest of 980 hectares of diverse forest landscape and 50 km of forest road.

The slope has a southwesterly aspect, with an average inclination ranging from 5 to 20%. The site covers an area of approximately 1 hectare and includes a forest road that provided access to the testing area for evaluating robots equipped with LiDAR scanning technology. The soil at the site is classified as nutrient-rich brown earth.

The stand is well-structured and has reached the developmental stage of “establishing pole wood.” Additionally, a partially extensive natural regeneration layer comprising spruce (Picea abies) and fir (Abies alba) has developed beneath the main canopy. In this two-layered stand, canopy closure varied between open and closed.

The species composition in the overstory is as follows:

Spruce (Picea abies): 60%

Pine (Pinus sylvestris): 20%

Fir (Abies alba): 10%

Beech (Fagus sylvatica): 10%

**Fig. 1:**
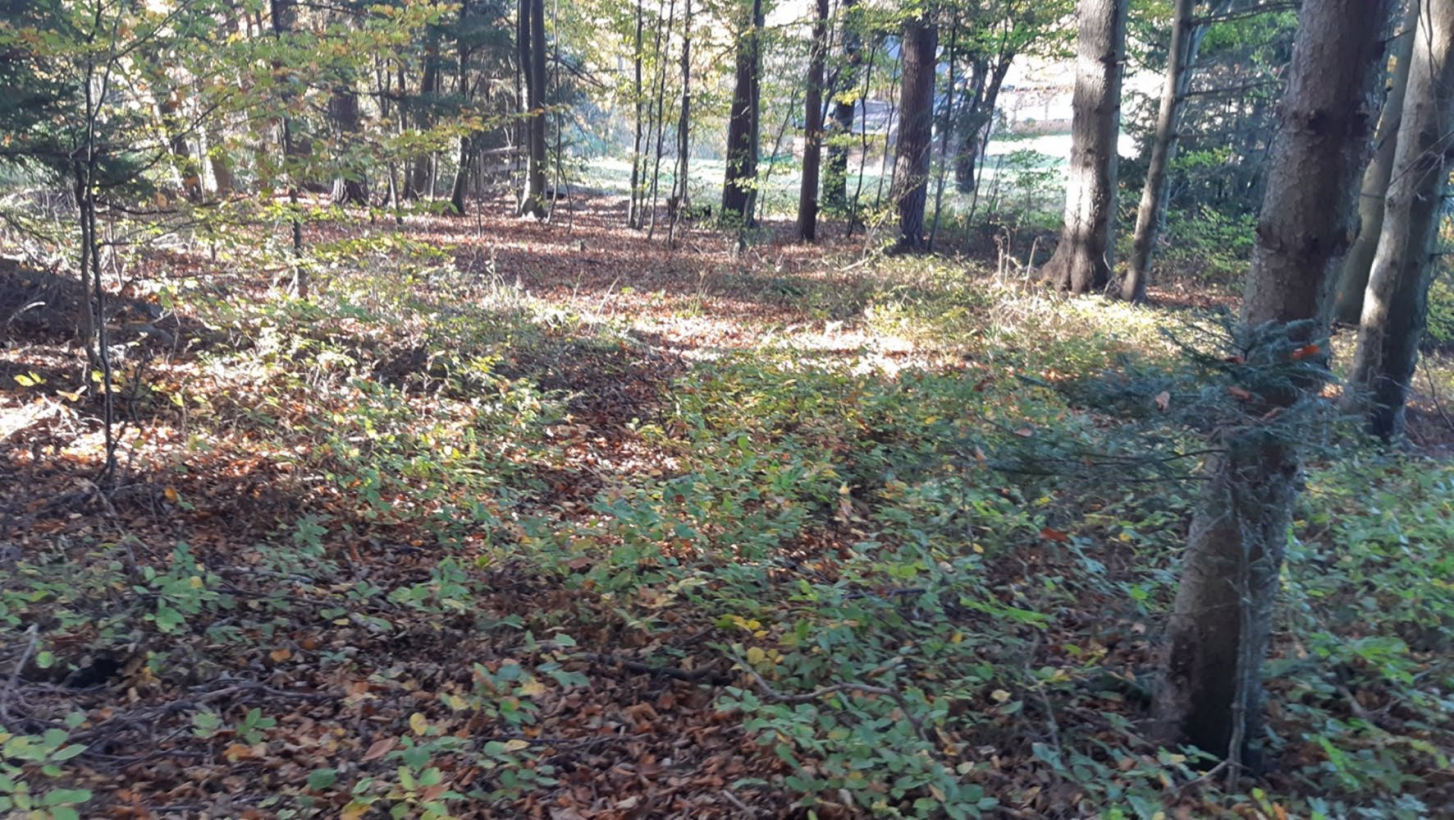
Our Rosalia Teaching Forest–with 980 hectares of diverse forest landscape the “largest human-centered AI Lab of the world”. A view of the testing area: a very complex and challenging environment for robots

The initial conditions for the driving test were favorable, with dry soil partially covered by beech litter and sunny autumn weather at 15 °C (Fig. 1).

### Participants

The testing group consisted of 27 forest engineering students. All of them were in the age bracket of 22 to 26 years old. The students were all at the end of their Bachelor term knowing and understanding the forest quite well compared to an untrained professional but still not at the level of a seasoned expert. The students were always deployed in groups of two, but the tested robot was changed for each participant to avoid the second student learning from following the first one. The handheld LiDAR scanning was performed by each of the pair.

### Robot testing setup in the wild

The testing setup involved three robotic platforms: Boston Dynamics Spot, AgileX Scout, and AgileX Bunker Mini. Each robot was equipped with an Ouster LiDAR scanner, along with a battery and laptop to support the system. The experiment aimed to evaluate the performance of these robots in challenging forest environments, focusing on their maneuverability, stability, and robustness.

### Robot platforms

#### Boston dynamics spot

**Fig. 2:**
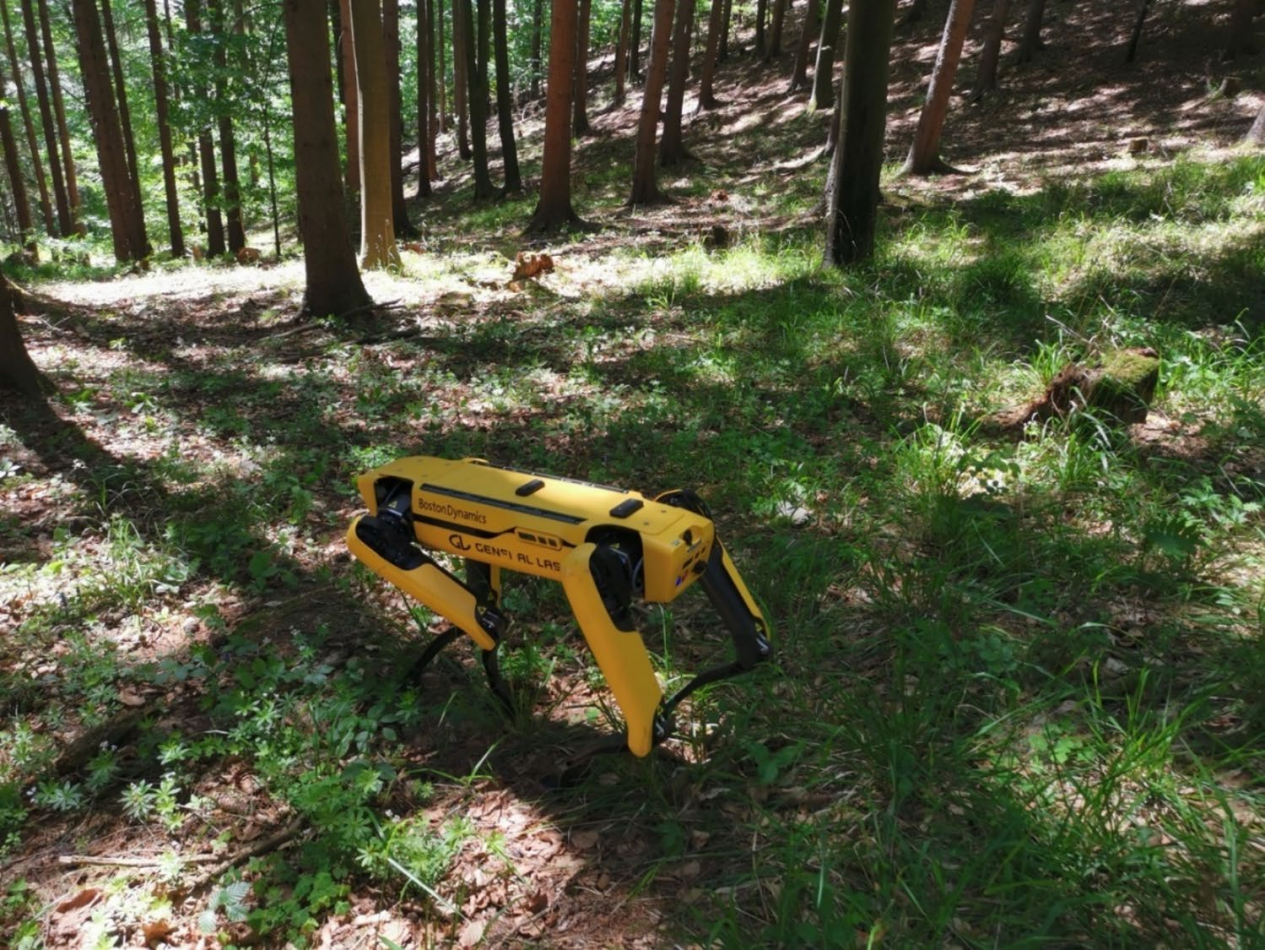
Boston Dynamics’ Spot traverses a dense forest, encountering challenges posed by dense undergrowth and uneven terrain. The combination of fallen branches, exposed roots, and thick vegetation presents a formidable obstacle for legged locomotion. This scenario underscores the need for improved sensor fusion, adaptive gait strategies, and AI-driven terrain awareness to enhance robotic deployment in forestry applications

The Boston Dynamics Spot is a quadrupedal robot designed for highly agile and versatile locomotion. It is particularly effective on uneven terrain due to its dynamic balancing capabilities (Fig. 2).

#### AgileX scout

**Fig. 3:**
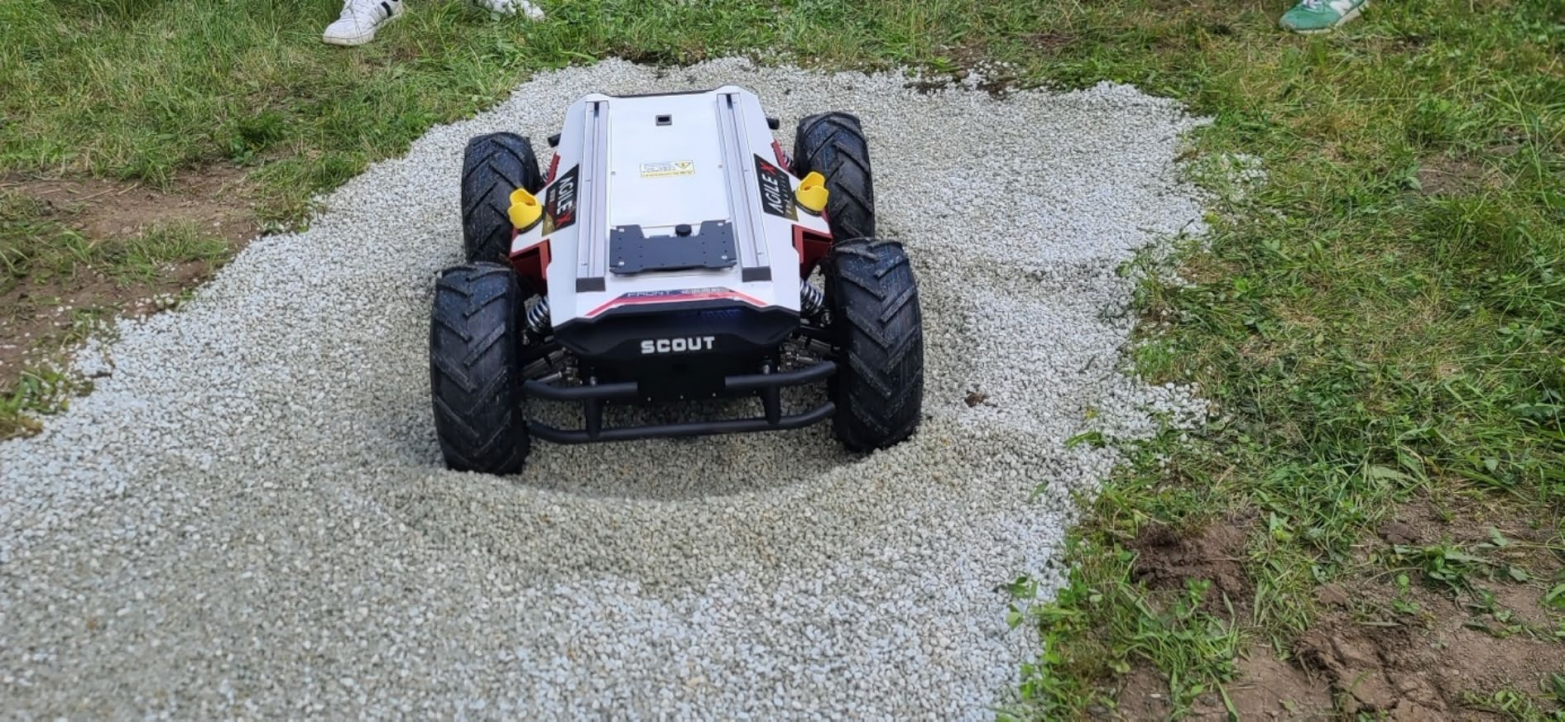
AgileX Scot navigates a gravel pit: Loose, uneven gravel and shifting slopes pose significant traction and stability challenges, testing the vehicle’s skid-steering dynamics and suspension system. Despite its low center of gravity and rugged wheels, the Scout experiences wheel slippage and localized sinkage, requiring real-time adjustments in throttle and steering to maintain maneuverability. This controlled experiment highlights the strengths and limitations of wheeled robotic platforms in deformable terrain, emphasizing the need for improved terrain adaptation algorithms, dynamic traction control, and predictive path planning for autonomous deployment in unstructured environments

The AgileX Scout is a compact, rugged, wheeled and robust all-terrain UGV (Unmanned Ground Vehicle) used as robot designed for outdoor applications, capable of handling moderately rough terrain with ease (Fig. 3).

#### Agile X bunker mini

**Fig. 4:**
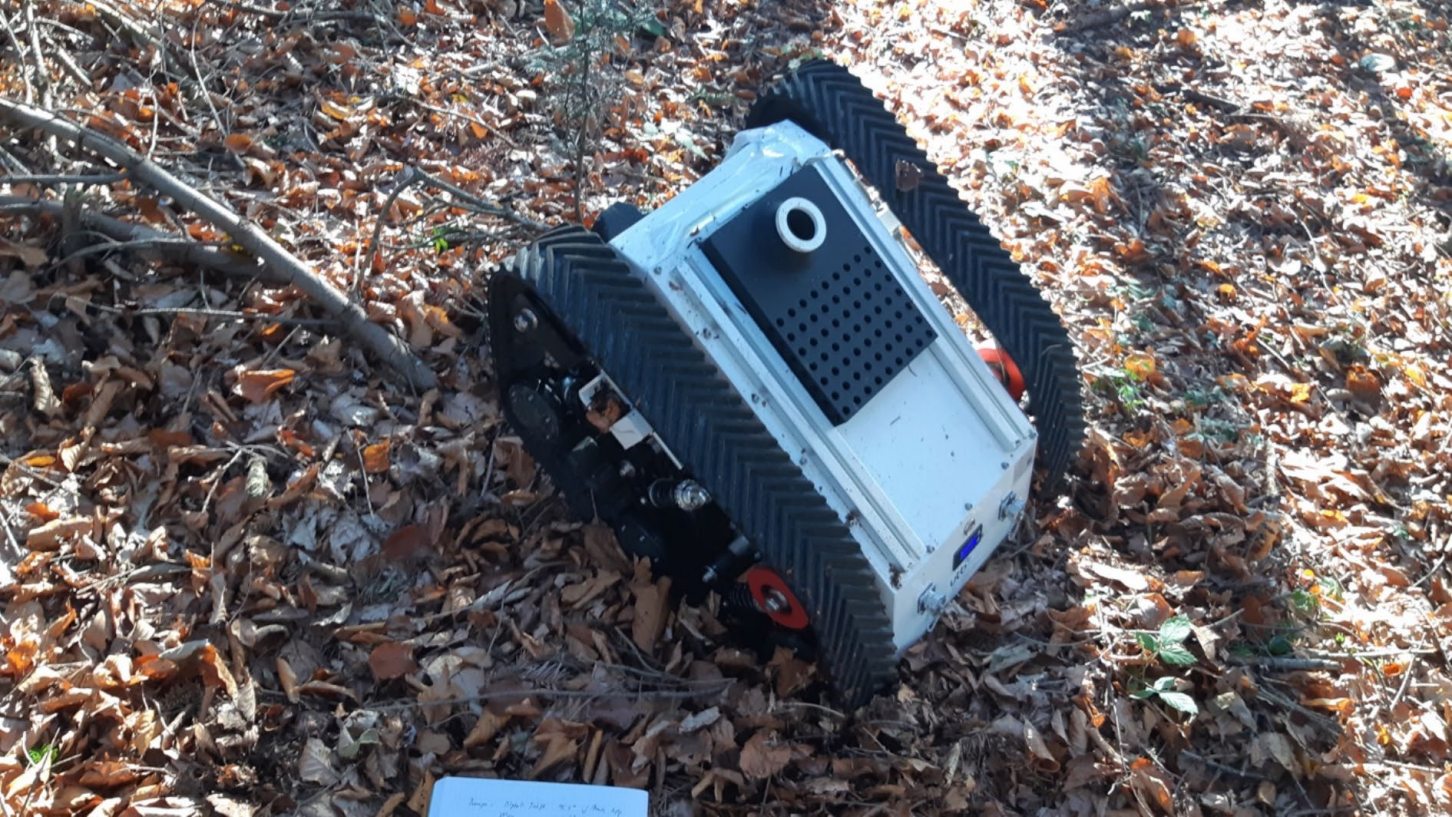
AgileX Bunker Mini—operational in the forest and equipped with a Lidar Scanning-Adaptor offers excellent traction and resistance to tipping

A tracked robot designed for high stability and robust operation in extreme environments. It offers excellent traction and resistance to tipping. The AgileX Bunker Mini, a rugged, tracked UGV, operates in a dense forest environment, equipped with a LiDAR scanning adaptor for enhanced perception and autonomous navigation. The tracked platform provides superior traction over soft soil, roots, and uneven terrain, allowing it to traverse challenging landscapes where wheeled robots might struggle. The onboard LiDAR continuously maps the surroundings, detecting obstacles such as fallen branches and dense undergrowth, while also aiding in real-time SLAM (Simultaneous Localization and Mapping) for precise localization. Despite its robust mobility, the robot faces challenges such as LiDAR occlusions from dense foliage and variations in terrain reflectivity, which can affect sensor performance. This setup highlights the interplay between mobility and perception in robotic forestry applications, emphasizing the need for sensor fusion strategies and adaptive autonomy in unstructured environments (Fig. 4).

### Testing conditions

The robots were tasked in the wild with navigating three distinct forest terrains:

- Forest road: A relatively even but occasionally uneven dirt path;
- Regeneration stands: Areas with young, regenerating trees requiring careful manoeuvring to avoid damage;
- Open forest stands: Regions with leaf-covered ground, interspersed with partially decayed wood and deadwood obstacles.

Each robot was tested under two configurations:

- With LiDAR payload: Including the attached Ouster LiDAR scanner, battery, and laptop;
- Without LiDAR payload: To identify inherent failure points in the robot's design and performance without additional weight or hardware.

### Procedure

The robots were operated by untrained forest engineering students who received a brief 5-min introduction to key operational factors, including:

- Identifying tipping points;
- Avoiding damage to regenerating forest stands.

After the introduction the students were tasked to first follow a well-maintained forest road with an average inclination of 7% for 350 m to enter the forest stand. Two areas were defined. One open area with minimal regeneration or dead wood which participants navigated for 30 min and then moved into denser terrain with more regeneration and dead wood and closer trees. In this area they spend 1 h. They were tasked to cover the entire area as much as they could during this time ensuring that each participant had to maneuver into challenging terrain. Each of the testing areas had an area of 0.5 hectare and resembled an almost perfect square. The boarders of the testing area were set before and communicated to each participant and participants were constantly monitored to not leave the testing area. Other than these restrictions students had the freedom to maneuver however they saw fit. This approach simulated real-world scenarios where operators may not have extensive training or experience with robotic platforms and a certain area of interest must be covered. The emphasis on the harsher terrain was used to push both robot and operator to their limits.

### Data collection and evaluation

Failures were monitored, detected, and evaluated across all platforms, with specific attention to:

- Stability issues (e.g., tipping over, getting stuck, and being unrecoverable by their own power);
- Performance degradation in different terrains;
- Interaction with the forest environment, such as damage to regeneration stands or difficulty navigating leaf-covered areas.

### System usability methods

A previous study investigated the usability of human–robot collaboration in the underexplored domains of forestry and agroforestry. The study utilized two robotic platforms: Boston Dynamics Spot, AgileX Bunker Mini and AgileX Scout. The study was conducted in the educational forest of BOKU and allowed the direct testing in a real forest environment, focusing on the human robot interaction. Usability was assessed using the System Usability Scale (SUS), a well-established metric in human–computer interaction.

The findings demonstrated the potential of these robotic systems to enhance productivity and safety, highlighting the importance of user-centered design in the development of collaborative tools. The study concluded that integrating AI-driven technologies into forestry and agriculture requires a human-centered AI approach, prioritizing usability, accessibility, and the principle of universal access.

This system usability study adheres to the international usability standard ISO 9241–11, which includes both objective and subjective evaluation metrics [42]. The System Usability Score (SUS) assesses users’ comfort and positive attitudes toward using the product. Satisfaction was measured through surveys or questionnaires completed by users after performing tasks, allowing for an evaluation of their subjective experience. In this study, the System Usability Scale was employed.

Tables 1 and 2 present the SUS questionnaire utilized in the experiments. Each statement was rated on a scale from 1 to 5, where 1 indicates the participant did not agree at all with the statement, and 5 indicates full agreement. For ease of comprehension the questions are located in the results section next to the answers.

**Table 1:** SUS questionnaire-LiDAR

No	Statement
1	I would regularly use the LiDAR scanner for data collection
2	I found the LiDAR scanner unnecessarily complex
3	The LiDAR scanner was easy to use
4	I think I would need assistance from a technician to efficiently use the LiDAR
5	The various functions of the LiDAR scanner are well integrated
6	I noticed inconsistencies in the operation of the LiDAR scanner
7	I think most people would quickly learn how to use the LiDAR scanner
8	The LiDAR scanner was cumbersome to operate
9	I felt confident using the LiDAR scanner
10	I needed to learn a lot before I could effectively use the LiDAR scanner

**Table 2:** SUS questionnaire-robots

No	Statement
1	I would like to use the robot regularly
2	I found the robot unnecessarily complex
3	The robot was easy to use
4	I think I would need assistance from a technical person to operate the robot
5	The robot's various functions are well integrated
6	I noticed inconsistencies in the robot's operation
7	I think most people would quickly learn how to use the robot
8	The robot was cumbersome to operate
9	I felt confident using the robot
10	I needed to learn a lot before I could use the robot effectively

The analysis was conducted using a range of statistical measures and tests provided by the Usability Statistics Packages tool, which is accessible online [43, 44]. To standardize the data, participants’ scores for each question were transformed from their original range of 0–40 to a normalized scale of 0–100. It is important to note that, while the resulting scores fall within the 0–100 range, they are not percentages in the conventional sense. Instead, these values are intended to be interpreted as percentile rankings, providing relative performance comparisons rather than absolute measures [45, 46].

### Physical exertion

**Fig. 5:**
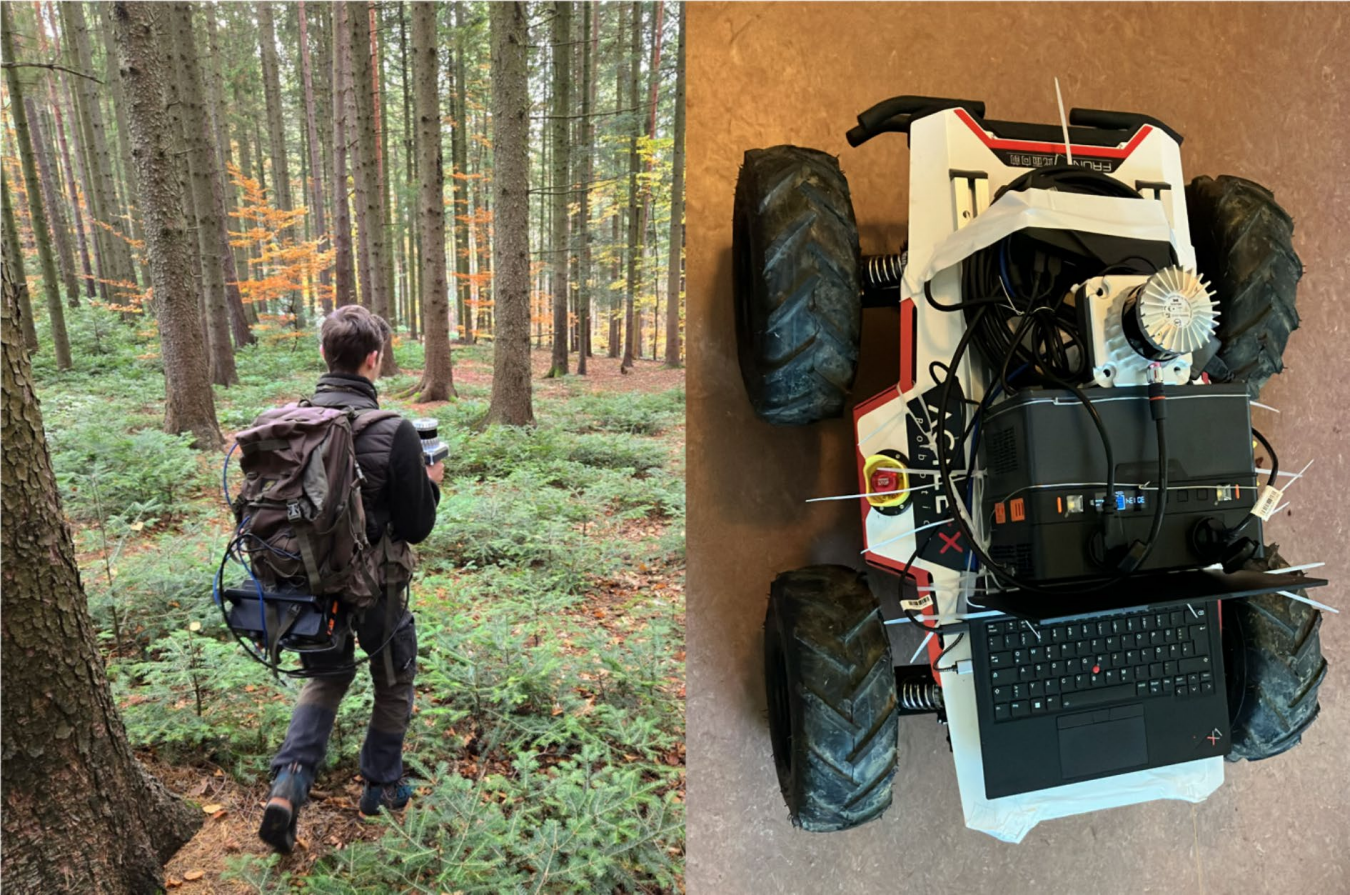
Left: Handheld LiDAR Scan, the Scanner is visible in the left hand and all electronics components are transported in the backpack; Right Robot equipped with LiDAR Scanner

This basic test was conducted to evaluate the physical workload experienced by operators performing LiDAR scanning tasks under two different configurations. In the first method, participants carried and manually operated a handheld LiDAR scanner. In the second method, they remotely maneuvered a robot equipped with the same scanner. The objective of this study was to assess the potential workload reduction for human operators when robotic assistance is utilized for LiDAR-based forest scanning tasks. Figure 5 highlights the setup.

Before the tasks, participants completed a questionnaire assessing their physical readiness and pre-existing discomfort or fatigue. They rated their preparedness on a scale from 1 to 10 and indicated whether they felt muscle soreness, stiffness, or tiredness. Additionally, they identified environmental conditions such as heat, cold, humidity, rain, or rough terrain that they anticipated might affect their physical performance.

After completing the tasks, participants provided feedback on their physical state and compared their post-task exhaustion levels to their initial state. They rated their physical exhaustion on a scale from 1 to 10 and described any changes in muscle soreness or joint pain. The questionnaire also asked about the influence of terrain and environmental factors on their energy levels, the effectiveness of breaks in managing fatigue, and whether carrying or handling the equipment contributed to their fatigue. Participants were further asked whether physical fatigue impaired their concentration or sense of safety and identified the most physically challenging aspect of the tasks through an open-ended question. The questions are available in Table 3, located in the results section for ease of comprehension.

**Table 3:** Physical exertion questionnaire

No	Question
1	On a scale of 1 to 10, how physically prepared do you feel right now for the upcoming tasks? (1 = Not prepared at all, 10 = Very well prepared)
2	Do you currently feel muscle soreness, stiffness, or fatigue? (Not at all, Slightly, Moderately, Clearly)
3	Which environmental conditions do you think could impair your physical performance today? (Multiple choice: Heat, Cold, Humidity, Rain, Rough terrain)
4	On a scale of 1 to 10, how physically exhausted do you feel after completing the tasks? (1 = Not exhausted at all, 10 = Extremely exhausted)
5	Rate the level of muscle soreness or joint pain compared to before the tasks began. (Decreased, No change, Slightly increased, Clearly increased)
6	How would you describe the impact of the terrain and environment on your energy levels during the tasks? (Low, Moderate, Significant)
7	How effective were any breaks in managing your fatigue? (Not effective, Somewhat effective, Very effective)
8	Did you feel that carrying or handling equipment contributed to your fatigue? (Not at all, Slightly, Moderately, Clearly)
9	Did physical fatigue affect your concentration or sense of safety during the tasks? (Not at all, Slightly, Moderately, Clearly)
10	What do you think was the most physically challenging aspect of the tasks completed today? (Open answer)

The data collected was analyzed to compare the physical demands of handheld LiDAR operation with those of a robot-mounted system. Key areas of focus included baseline physical state versus post-task exhaustion, the impact of environmental conditions, and the role of equipment handling in contributing to fatigue. As a preliminary evaluation, this test provided foundational insights into how robotic systems can help reduce physical workload.

## Results

### Robot movement

The three tested robotic platforms exhibited significant differences in their ability to navigate various terrains. The following table outlines each of them separately. Key discussion points include operational time, overall performance across the entire testing terrain, distinct failure points, and environmental impact (Table 4).

**Table 4:** Comparison of different robotic platforms for their use in forest applications

Robotic system	Operational time	Terrain performance	Failure points	Environmental damage
Boston dynamics spot	30 min–50 min	Excellent on areas without dense vegetation—only solid surfaces	Regeneration and dead wood/branches on the ground	No damage detectable
Agile X Scout	180 min—300 min*	No major restrictions	Dense regeneration and objects larger than 35 cm**	Damage on regenerative trees at high speeds
Agile X Bunker Mini	130 min—270 min*	Excellent on flat surfaces	Regenerative trees and obstacles between 17 and 25 cm height**	Destruction of bark on regenerative trees by the free-spinning tracks

^*^Overall time estimated from battery consumption during the test procedure, both AgileX robots were car*rying the Lidar Scanning setup*

^**^Sloped objects are easier to overcome compared to relatively steep ones like a log

**Fig. 6:**
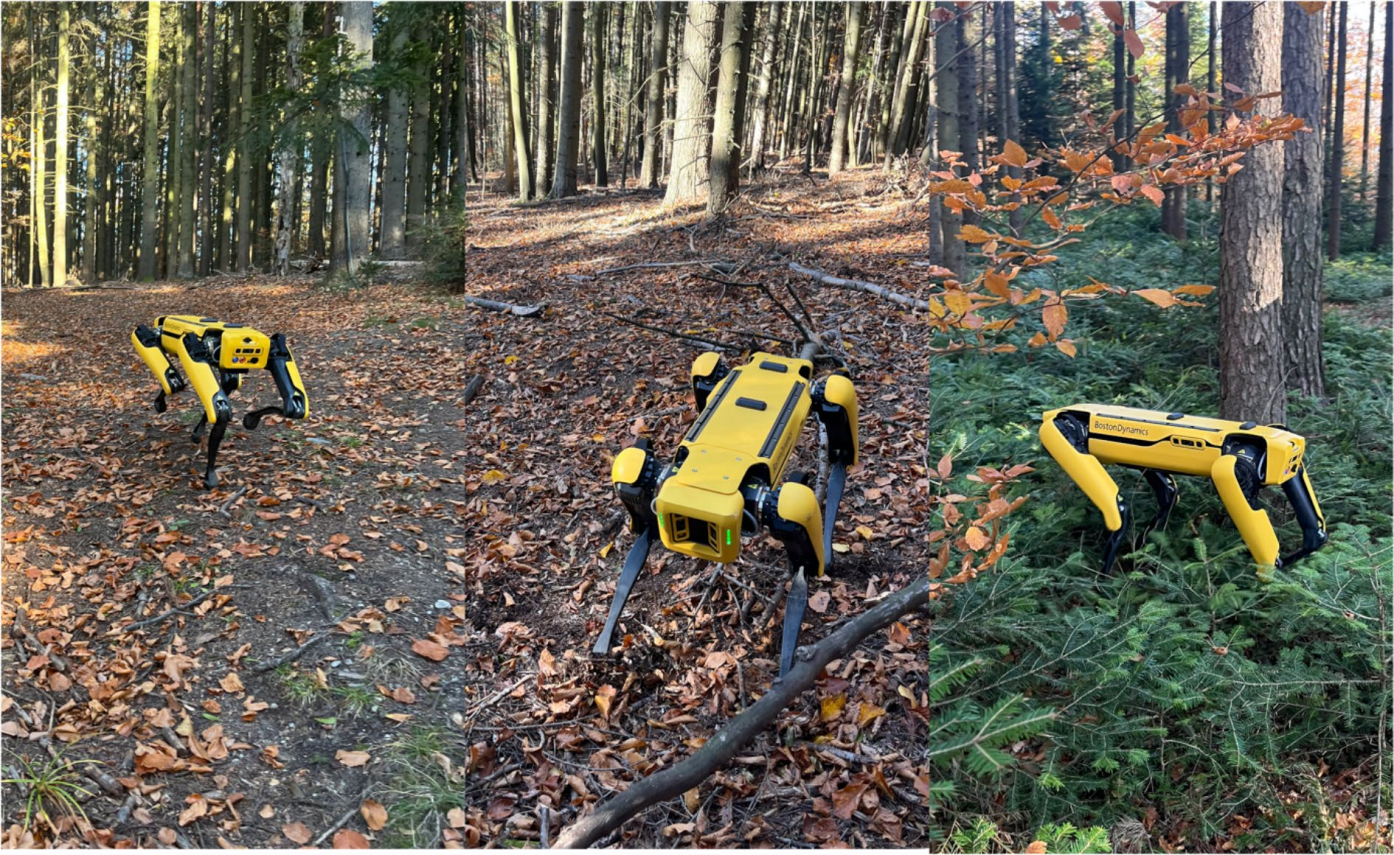
Boston Dynamics in different forest terrains. Left simple no operational problems; Center partially problematic depending on log size and position; Right Spot will fall over and a reset is required

Initial expectations were high for Boston Dynamics Spot. Its quadrupedal locomotion system intuitively makes the most sense for complex environments [47], and it represents an evolution in what a robot can do and how it looks [48]. However, its performance in the forest painted a different picture. Solid surfaces, regardless of inclination, and obstacles on such solid surfaces -such as logs- can be easily overcome as the robot simply steps over them. The situation changes drastically when operating in areas with uncut grass or young trees (Fig. 6).

The sensors of the robot do not allow it to clearly determine where to step, often resulting in frantic movements that end with the robot toppling over. After such a failure, the robot must reboot itself, requiring an operator to maneuver it into an open, relatively flat area. This resetting process can take up to 15 min and is physically demanding, as the robot weighs 33 kg. Due to its tendency to topple over, no LiDAR scanner was mounted on it as a safety precaution. Another major downside is the battery runtime, which ranges from 30 to 50 min—far from sufficient for any forest operation, primarily due to the lack of recharging options. The main advantage Spot could offer, if the maneuvering issue in areas with young trees and high grass is resolved, is its minimal impact on vegetation. This was expected, as its movement pattern more closely resembles that of an animal.

The more conventional setup of AgileX Bunker Mini and Scout proved to be more functional, as their underlying technologies have been in use for a long time. Movement on forest roads and flat forest areas was completely unproblematic. However, turning on softer surfaces disturbed the ground conditions. While this may not be highly problematic for smaller robots, it is definitely a factor that must be considered [49].

**Fig. 7:**
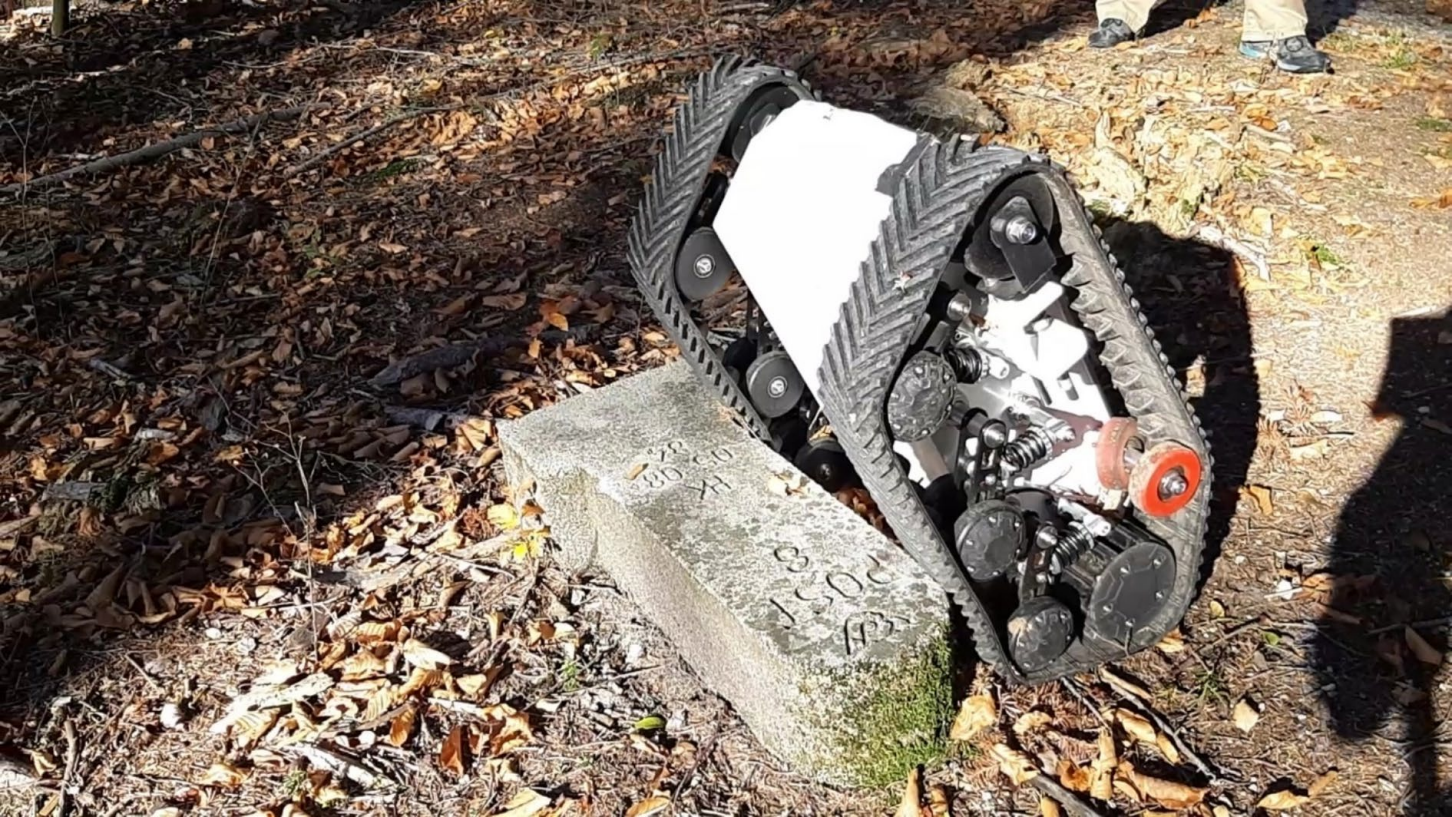
Tipping issue of AgileX Bunker Mini while tackling an obstacle

The major limitations surfaced when attempting to maneuver a predefined grid that a human operator could easily follow but could not be replicated in the same way by either robot. Logs and obstacles that can be stepped over with ease by a human often presented insurmountable barriers for the robots. The tracked Bunker Mini, in particular, faced issues quickly—when attempting to climb an obstacle, its pivot point shifted to the back of the robot, making it difficult to maintain stability (Fig. 7).

**Fig. 8:**
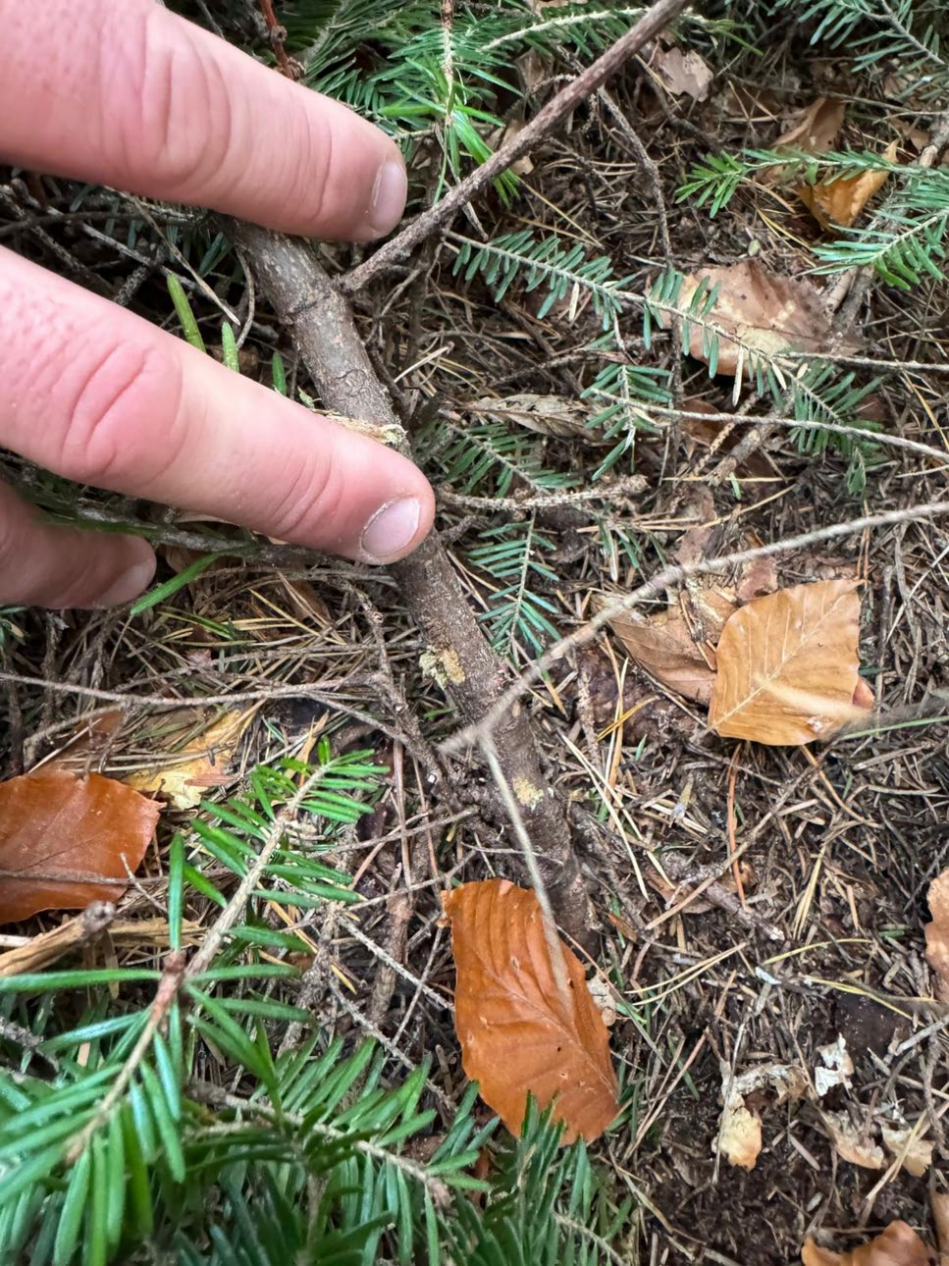
Environmental Impact of Robotic Mobility: Visible damage to a young tree following the passage of the Bunker Mini highlights the importance of designing robotic platforms that minimize ecological disturbance. This observation underscores the need for environmentally sensitive locomotion strategies in forest robotics, ensuring that automation supports forestry operations without compromising ecosystem integrity. Future developments should prioritize adaptive mobility systems that balance efficiency with environmental responsibility in complex natural terrains

This shift in pivot points limits the robot’s ability to overcome obstacles taller than 25 cm. Maneuvering through dense vegetation is possible due to the robot's weight, which allows it to push over young and soft vegetation. However, under these conditions, the tracks frequently lose traction and strip the bark off young trees without achieving any forward movement, resulting in unintended damage [50] (Fig. 8).

AgileX Scout, with its four-wheel-drive setup, demonstrated the best performance under the varied conditions encountered in this test. The operational time of approximately five hours under full load is the closest to a viable system for forest applications. It is also important to note that all platforms were intentionally pushed to their limits, consuming more power than they would under standard operating conditions. In this study all the robots were tackling the worst possible terrain that could be encountered on purpose and the time spend in such terrain was much higher than expected in a standard operation. Not only because most company owned forests look similar to Fig. 6 on the left, but also due to the fact that the route taken for a scanning operation is preset and only a small amount would normally fall under extreme terrain. It has to be mentioned that the same principle of worktime applies to human operators, in complex terrains the human requires more breaks, and the effective worktime is reduced. AgileX Scout performed well in all tested scenarios, with its only limitation being obstacles higher than 35 cm.

Its main advantage lies in its wheeled setup, which influences the pivot point, preventing it from tipping over. Additionally, when one wheel passed over an object, the center of gravity of the entire robot was immediately lowered as the obstacle was caught between the two wheels. The primary limitation, similar to other platforms, is maneuvering in dense regenerative forests. However, damage to young trees was minimal and could be entirely avoided by covering the bottom edges of the robot, which were the only points that caused minor vegetation damage.

Overall, simple wheeled systems appear to perform best in these complex environments. Systems like Boston Dynamics Spot have the potential to enable even greater maneuverability if two critical challenges are addressed: first, improving operational time, and second, developing software-based solutions that allow the robot to function effectively in areas with dense vegetation. Initial progress has been made in these areas, but further development is still required [51].

### Physical exertion

To provide a real-world comparison for a specific forestry task, LiDAR scanning for forest inventory was employed. The handheld LiDAR scan served as a baseline, as it is an established technology for forest inventory estimations. In a forestry setting, this type of LiDAR scanning is referred to as Personal Laser Scanning [52].

The testing location was simple enough to ensure that all participants could easily maneuver the equipment without encountering physical limitations that could not be overcome. The questionnaire on physical exertion provides only a general indication of how physical workload differs between Personal Laser Scanning and Robot-Assisted Laser Scanning. However, a more in-depth understanding would require additional testing.

The first three questions of the questionnaire were designed solely to determine the physical state of each participant before the experiment began. All responses are available in Tables 5 and 6.

**Table 5:** Physical exertion answers-LiDAR handheld

No	A	B	C	D	E	F	G	H
1	10	10	10	7	5	8	8	8
2	Not at all	Not at all	Moderately	Not at all	Not at all	Not at all	Slightly	Slightly
3	Heat, rain, rough terrain	All answers	Rough terrain	Heat, rough terrain	None	Heat, rain	Rough terrain, rain	Rain, terrain
4	1	1	2	5	3	2	2	2
5	No change	No change	Slightly increased	Slightly increased	No change	No change	No change	No change
6	Significant	Moderate	Low	Moderate	Significant	Low	Moderate	Significant
7	Not effective	Very effective	Not effective	Somewhat effective	Not effective	Very effective	Somewhat effective	Somewhat effective
8	Not at all	Slightly	Moderately	Not at all	Moderately	Moderately	Significantly	Significantly
9	Not at all	Slightly	Not at all	Not at all	Not at all	Moderately	Not at all	Moderately
10	Walking		Carrying LIDAR through the forest	Carrying LIDAR through the forest	Different obstacles and long time are problematic		Carrying the LIDAR is problematic

**Table 6:** Physical exertion answers-robot

No	A	B	C	D	E	F	G	H	I
1	10	10	10	10	9	10	10	8	10
2	Not at all	Moderately	Not at all	Moderately	Slightly	Moderately	Not at all	Slightly	Moderately
3	All	Rough terrain, rain	Heat, terrain	Cold	Heat, rain	Heat, rough terrain	Heat, terrain, cold	Rough terrain	Humidity, heat
4	3	7	3	2	1	-	3	4	8
5	No change	Slightly increased	Slightly increased	Decreased	No change	No change	No change	Slightly increased	Slightly increased
6	Significant	Significant	Significant	Moderate	Significant	Low	Moderate	Significant	Significant
7	Somewhat effective	Very effective	Very effective	Very effective	Very effective	Not effective	Very effective	Somewhat effective	Somewhat effective
8	Moderate	Moderate	Slightly	Moderate	Slightly	Not at all	Significant	Significant	Not at all
9	Moderate	Not at all	Not at all	Slightly	Moderate	Slightly	Slightly	Slightly	Moderate
10	Recovery of the defective robot	Carrying Spot through the forest	-	Walking in the long term is exhausting	Finger issues with joysticks – finger relaxation	Strain comes from constant focus on the robot	Recovering the robot	Carrying/raising the robot	None

It is important to note that carrying the LiDAR setup through the forest influenced participants' perceived readiness to perform the task. While using the robot did not lead to any anticipation of physical exertion, carrying the LiDAR scanner resulted in noticeable physical awareness of the task. The factors of soreness and fatigue showed a consistent tendency across both groups, with all participants experiencing slight fatigue. However, when considering the most concerning factors during the study, no clear trend emerged. Some participants were aware of all factors, while others focused only on two or three. Overall, environmental conditions were a significant concern for all participants [53].

The following section presents the results after the actual test. Overall, exertion levels after the experiment were relatively low, though there was a noticeable spike in the robot test group compared to the PLS group. This is likely an outlier, as the group operating Spot had to carry the heavy robot multiple times to reset and restart it. In general, physical exertion remained low, and participants did not report any significant increase in exertion compared to their initial state. To better understand this factor, testing in a more physically demanding environment with greater inclination would be necessary. Both groups recognized that environmental conditions play a crucial role in the difficulty of the task. Similar to the effect of the environment, all participants agreed on the effectiveness of breaks in managing fatigue [54]. Additionally, handling the equipment was a critical factor influencing exertion across all groups, and loss of focus due to fatigue was reported at similar levels in both test groups.

The open-ended question at the end of the study was used to identify potential points of exertion unique to each setup. Three key issues were highlighted by the robot group. The first was robot recovery, which, as mentioned earlier, could likely be mitigated by selecting the appropriate robot for the task and ensuring operators receive proper training to reduce the need for recovery. The second concern was long-term effects, such as fatigue from excessive walking, which led to a loss of focus. The third issue, from an ergonomic perspective, was finger pain experienced by one participant due to the fine control adjustments required for the robot’s remote control. This problem could be addressed by modifying the control setup of the robots. For the LiDAR scanner, the primary concern was carrying the equipment. Additionally, increased operation time in more challenging terrain, such as deadwood and other obstacles, could make the task more physically demanding.

### System usability scale

The core focus of this publication is the system usability in its actual environment of use. A total of 28 participants tested each setup and completed the SUS questionnaire. Before analyzing the results, it is important to note that some responses showed a high correlation with one another, suggesting that merely separating participants while filling out the questionnaire was not sufficient to prevent cross-influence. Therefore, for future studies, it is recommended that each participant completes the questionnaire individually without any potential overlap with others. The studies are divided into four parts—three covering the robotic platforms and one focusing on handheld LiDAR operation. This section begins with the discussion of handheld LiDAR. With a score of 78 ± 6 on the SUS scale, its usability can be classified as "Good." This indicates that the system is practical for the operator and does not present significant drawbacks. This value will serve as the benchmark for evaluating the usability of the robots (see Table 7).

**Table 7:** SUS results-Handheld LiDAR

Qs Nr	A	B	C	D	E	F	G	Mean
1	5	5	5	3	4	5	4	4
2	1	2	4	2	2	2	2	2
3	5	4	5	4	4	4	4	4
4	3	3	4	3	2	3	1	3
5	4	5	3	4	4	3	4	4
6	1	3	1	3	4	2	3	2
7	4	5	4	4	5	4	4	4
8	1	2	1	3	1	1	1	1
9	5	5	5	5	4	5	5	5
10	3	2	1	3	1	2	2	2
SUS Pos	23	24	22	20	21	21	21	22
SUS Neg	9	12	11	14	10	10	9	11
SUS Total	85	80	78	65	78	78	80	78 ± 6

The most promising candidate in terms of usability from previous studies was Boston Dynamics Spot. However, testing in a more complex environment, beyond a specially designed usability test course, revealed significant issues for forest applications. The overall SUS score was 78 ± 10, presented in Table 8. This result is very close to that of handheld scanning, but the higher standard deviation indicates that the system does not provide a consistent user experience for all operators.

**Table 8:** SUS results-boston dynamics spot

Qs Nr	A	B	C	D	E	F	G	Mean
1	5	4	4	4	5	3	5	4
2	1	2	1	1	4	2	2	2
3	5	5	4	5	4	4	5	5
4	1	2	1	2	2	2	1	2
5	5	3	3	2	3	2	2	3
6	2	4	5	4	3	5	4	4
7	5	5	5	5	5	5	4	5
8	1	2	2	1	1	2	1	1
9	5	5	5	5	2	5	5	5
10	1	2	1	1	1	1	1	1
SUS Pos	25	22	21	21	19	19	21	21
SUS Neg	6	12	10	9	11	12	9	10
SUS Total	98	75	78	80	70	68	80	78 ± 10

Considering the frequent failures due to Spot tipping over and the potential damage to the LiDAR scanner, the SUS score does not justify Spot as a viable alternative or addition to conventional Personal Laser Scanning. This deviation from previous studies suggests that Spot is optimized for specific terrains and lacks built-in adaptability for varying environments. Therefore, Spot would perform better in a forestry setting if reprogramming were conducted on the developer’s side. However, as this application remains a niche use case, rapid development in this area is not expected.

The two simpler platforms in the form of AgileX Bunker mini (88 ± 5, Table 9) and AgileX Scout (88 ± 4, Table 10) showed a clear increase in the SUS score. Both at least partially enter the realm of "Excellent" usability. Both systems show low standard deviation, and all users were able to operate them without major challenges. The small change in standard deviation is probably the effect of Bunker Mini to get entangled in vegetation easier and additionally the tendency to tipple over while crossing obstacles making it more demanding for the operator. The significant increase in the SUS score suggests that operating Personal Laser Scanning with robot assistance can immediately enhance the Usability of this specific forest operation. An interesting correlation between usability and SUS score can be drawn in the degrees of freedom of movement for each robotic platform. Scout and Bunker both only allow 2 degrees of freedom, back and forth as well as rotation around the central axis, while Spot allows way more complex movements. The assumption here is that less degrees of freedom make the understanding of how the system operates easier to understand for the user. It is very straight forward to have forward and backward motion and a way to turn left or right, whereas Boston Dynamics allows for many more manipulations ranging from moving sideways to adjusting height. These factors are probably something the operator can get used to over time and the differences will be minimal but what cannot change is the complexity of the system itself. The complex movement pattern of Boston Dynamics relies on complex sensor and camera input combined with a lot of computing to combine all the inputs into a motion that results into motion. This complexity showed in the complex environment and yielded in many failures that were not caused by the operator but the robot itself. Therefore, less degrees of freedom seem to be better for highly complex environments. Nevertheless, if the system reliability of Boston Dynamics increases it would be a better suited option for forestry tasks, but more degrees of freedom lead to a more complex and failure prone system.

**Table 9:** SUS results AgileX Bunker-Mini

Qs Nr	A	B	C	D	E	F	G	Mean
1	3	5	5	4	4	2	4	4
2	1	1	1	1	1	1	1	1
3	5	5	5	5	5	5	5	5
4	2	3	1	1	1	1	1	1
5	4	5	3	4	4	2	3	4
6	1	3	3	3	1	3	2	2
7	5	5	5	5	5	5	5	5
8	1	1	1	1	1	1	1	1
9	5	5	1	5	4	5	5	4
10	1	1	1	1	1	1	1	1
SUS Pos	22	25	19	23	22	19	22	22
SUS Neg	6	9	7	7	5	7	6	7
SUS Total	90	90	80	90	93	80	90	88 ± 5

**Table 10:** SUS results—AgileX scout

Qs Nr	A	B	C	D	E	F	G	Mean
1	2	2	2	4	3	4	4	4
2	1	1	1	1	2	1	1	1
3	5	5	5	5	5	5	5	5
4	1	1	1	1	1	1	1	1
5	3	4	3	3	4	3	3	3
6	2	2	4	3	3	3	3	3
7	5	5	5	5	5	5	5	5
8	1	1	1	1	1	1	1	1
9	4	5	4	5	5	5	5	5
10	1	1	1	1	1	1	1	1
SUS Pos	19	21	19	22	22	22	22	22
SUS Neg	6	6	8	7	8	7	7	7
SUS Total	83	88	78	88	85	88	88	88 ± 4

## Discussion

Moving from an artificial testing setup into the actual operational environment highlighted several important points. On one hand, it provided insights into where robots can excel, but it also revealed necessary adjustments to ensure proper performance. The most significant and controversial outcome of this study is the inability of Boston Dynamics Spot to function effectively in a forest environment. This limitation arises from multiple factors, starting with highly unpredictable maneuvering and frequent failures. Additionally, the SUS scores do not indicate an improvement in usability compared to existing setups, and Spot's overall usability decreased relative to previous studies. Finally, open-ended responses emphasized the challenges of maneuvering Spot after a failure to allow it to reset. This issue alone renders the robot unsuitable for regular forestry operations. Given these findings, we cannot currently recommend Boston Dynamics Spot for forest applications. However, as the system receives continuous updates, we will continue testing it in the future to assess potential improvements. In particular, enhancements in overcoming dense vegetation could make it a valuable tool for forest applications.

AgileX Scout and Bunker Mini performed well, and both appear suitable for the task at hand. From a SUS perspective, both setups are equally effective, achieving usability ratings in the excellent range. The primary differences emerged as operational limitations identified during testing. One key issue is the effect of tracks on the pivot point of the robot—a clear advantage for Scout, which was evident in multiple scenarios. A notable comparison point is each platform’s ability to move onto obstacles. Bunker Mini’s tracks allow it to push up nearly any obstacle, which in turn triggers issues related to its moveable pivot point. Conversely, the wheeled Scout does not face this problem, as its wheels do not provide enough traction to overcome very large obstacles, serving as a built-in failsafe against tipping over. The locomotion systems in both robots performed well across all tested surfaces, but increased damage to young regenerating trees was observed due to track slippage, which stripped bark. Considering these factors and the extended battery life of Scout, four-wheel-drive robots appear to be the most optimal off-the-shelf solution for forestry applications.

**Fig. 9:**
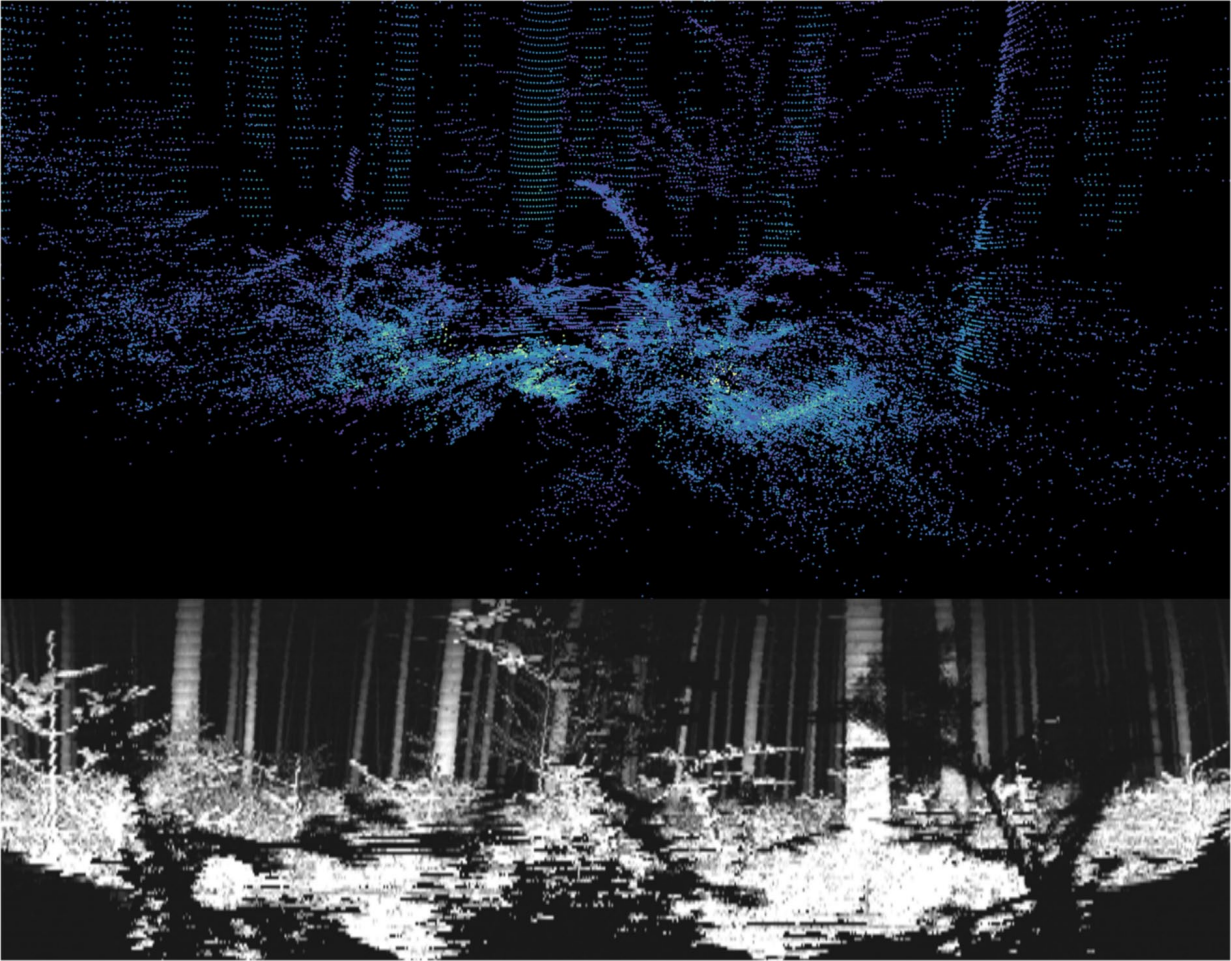
Top-LiDAR scan in regenerative trees; bottom-infrared image of the same view

Finally, the impact of the robot on the actual laser scanning task cannot be overlooked. When the LiDAR scanner is positioned correctly, the robot itself is never visible in the scan. This reduces post-processing workload compared to human-operated scanners, where the operator must be removed from the final scan. Otherwise, the scan results showed no significant differences. However, for Terrestrial Laser Scanning, where the scanner is set up stationary for each scan, the robot could serve as a mobile tripod, reducing setup time between scans. Overall, the scanning process worked well, and SUS results suggest that operating the robot compared to using the scanner alone is the preferred option for most users (see Fig. 9).

The shortcomings of this study also have to be mentioned. These are mainly linked to the fact that LiDAR scans were produced to understand the quality of the data generated but it was not designed to achieve optimal scanning results. Another study would need to be conducted where a proper scanning procedure is followed to address the single task of scanning a forest lot.

The comparison of physical exertion between both setups could not be clearly determined. The assumption would be that PLS is the more physically demanding of the two operations. However, the test duration and selected location represented a forest without extreme inclinations or large areas that would have increased exertion. Additionally, the weather conditions were favorable for light physical activity. To fully understand the differences in various environments, further studies would be required.

The most significant advantage is that the robot can be remotely controlled, allowing the operator to avoid entering hazardous areas. This feature has the potential to enhance safety if widely adopted. Reduced movement in unstructured terrain typically leads to fewer accidents.

The Universal Access community can leverage autonomous robots capable of navigating difficult terrains to significantly enhance accessibility and inclusion. Such robots could support remote participation by providing immersive exploration experiences of challenging natural or historic sites for users with mobility impairments. They might facilitate inclusive ecological or archaeological research, enabling users to remotely operate robots to perform detailed environmental monitoring, species observation, or cultural heritage documentation. Additionally, these robots could assist in adaptive recreation, allowing universal participation in activities such as wilderness exploration, trail mapping, or guided interactive tours of remote or otherwise inaccessible areas, greatly broadening opportunities for diverse user groups.

When comparing the physical exertion data presented in Tables 5 and 6, several principal differences and similarities can be observed. A clear difference emerges: participants reported feeling generally more physically prepared and experienced less fatigue when operating the robot-assisted system compared to the handheld LiDAR setup. This likely reflects two factors. First, the robot-assisted task was inherently less physically demanding, as users were not required to carry the relatively heavy LiDAR scanner and associated electronics through rough terrain. Second, participants appeared more confident and familiar with the robot-assisted task after initial instruction, possibly because the task was perceived as easier to control and less ambiguous in execution. Despite slight exertion from walking or handling controls, the robotic setup consistently led to lower exhaustion and fewer reports of muscle soreness, suggesting that the reduced workload and clearer task structure contributed to a higher perceived readiness. This supports the interpretation that it was not just a reduced physical burden, but also a more straightforward user experience that made the robotic task feel less onerous overall.

## Conclusion and future outlook

This paper has demonstrated the potential for utilizing robotic platforms in forestry applications. The initial testing setup for forest inventory generation with 3D-LiDAR scanning serves as a strong starting point, as this technology is becoming increasingly important in the forestry industry [55]. Engaging with forest engineering students—who will become the next generation of forest experts—helps expose them to the topic and showcases the future potential of robotics in forestry, following the universal access approach. Traditional industries that are slow to adopt novel technology can only progress if the next generation has the opportunity to test novel concepts and technologies early on, allowing them to be considered for future practical implementation. Currently, a simple wheeled robot is the best option for locomotion in forestry, as it can be easily equipped with a variety of sensors [56]. These robots are readily available and significantly more affordable compared to quadrupedal robots like Boston Dynamics Spot.

This study has raised several more complex research questions that require further evaluation. The next step will involve a testing setup designed to accurately assess the physical exertion of each participant using measurable parameters such as heart rate and body temperature. To achieve this, a wearable sensor setup is necessary, and robotic control will be further developed for fully remote operation, reducing the physical effort required by the operator. Additionally, the variability of different terrains will be adjusted to include both easier and more challenging sections, allowing for a clearer understanding of where robots can provide the most assistance to humans.

As this field of research continues to grow alongside advancements in Artificial Intelligence, AI integration presents its own set of challenges [57]. Furthermore, developments in 6G wireless communication should open up new possibilities for forestry in general and, most importantly, for forest robotics [58, 59].

Evaluating the performance of robots for laser scanning of large areas—particularly with remote-controlled or even partially autonomous operation—is the next key step. This could enable scenarios where a single operator controls multiple robots simultaneously, significantly increasing area coverage. Additionally, cost factors must be analyzed to determine where expenses can be minimized and how such systems can be seamlessly integrated into the industry. The ultimate goal is to develop a system that reduces workload, enhances safety, and does so without imposing high additional costs on the industry, as affordability is a key factor for the adoption of new technology. An intriguing aspect to explore in this setup is the implementation of reinforced implementation intentions to help operators avoid repetitive mistakes, particularly in potentially hazardous situations [60].

Beyond technical feasibility, this study underscores the need for cost-effective solutions that align with industry constraints. Future research will explore scalable deployment models, ensuring that robotic systems enhance productivity and safety without imposing prohibitive costs. Furthermore, cognitive support mechanisms, such as reinforced implementation intentions, will be investigated to help operators minimize errors, particularly in high-risk environments by improving the human–robot communication [61].

To fully unlock the potential of robotic forestry, real-world operational testing is the next essential step. Only through field validation can these systems be fine-tuned to meet the practical demands of forestry professionals. By systematically addressing usability, efficiency, AI integration, and affordability, this research lays the groundwork for a future where intelligent, autonomous robots become indispensable tools in sustainable forest management worldwide.

## Data Availability

No datasets were generated or analysed during the current study.

## Abbreviations

6G: Sixth generation wireless technology
AI: Artificial intelligence
ISO: International organization for standardization
LiDAR: Light detection and ranging
PLS: Personal laser scanning
SUS: System usability scale
UA: Universal access
UGV: Unmanned ground vehicle
